# Comparative effectiveness of combined therapy inhibiting EGFR and VEGF pathways in patients with advanced non-small-cell lung cancer: a meta-analysis of 16 phase II/III randomized trials

**DOI:** 10.18632/oncotarget.12294

**Published:** 2016-09-27

**Authors:** Yongzhao Zhao, Huixian Wang, Yan Shi, Shangli Cai, Tongwei Wu, Guangyue Yan, Sijin Cheng, Kang Cui, Ying Xi, Xiaolong Qi, Jie Zhang, Wang Ma

**Affiliations:** ^1^ Department of Oncology, The First Affiliated Hospital of Zhengzhou University, Zhengzhou, China; ^2^ School of Medicine, Tongji University, Shanghai, China; ^3^ School of Economic and Management, Tongji University, Shanghai, China; ^4^ Department of Emergency, The Affiliated Huaian Hospital of Xuzhou Medical University and The Second Peoples Hospital of Huaian, Huaian, China; ^5^ Mental Health Institute of the Second Xiangya Hospital, National Technology Institute of Psychiatry, Key Laboratory of Psychiatry and Mental Health of Hunan Province, Central South University, Hunan, China; ^6^ Department of General Surgery, Nanfang Hospital, Southern Medical University, Guangzhou, China

**Keywords:** effectiveness, combined therapy, EGFR, VEGF, non-small-cell lung cancer

## Abstract

**Background & Aims:**

Combined therapy inhibiting EGFR and VEGF pathways is becoming a promising therapy in the treatment of advanced non-small-cell lung cancer (NSCLC), however, with controversy. The study aims to compare the efficacy of combined inhibition therapy versus control therapy (including placebo, single EGFR inhibition and single VEGF inhibition) in patients with advanced NSCLC.

**Materials and Methods:**

An adequate literature search in EMBASE, Cochrane Central Register of Controlled Trials (CENTRAL), American Society of Clinical Oncology (ASCO) and European Society of Medical Oncology (ESMO) was conducted. Phase II or III randomized controlled trials (RCTs) that compared effectiveness between combined inhibition therapy and control therapy in patients with advanced NSCLC were eligible. The endpoint was overall response rate (ORR), progression free survival (PFS) and overall survival (OS).

**Results:**

Sixteen phase II or III RCTs involving a total of 7,109 patients were included. The results indicated that the combined inhibition therapy significantly increased the ORR (OR = 1.59, 95% CI = 1.36-1.87, p<0.00001; I2 = 36%) when compared to control therapy. In the subgroup analysis, the combined inhibition therapy clearly increased the ORR (OR = 2.04, 95% CI = 1.60-2.60, p<0.00001; I2 = 0%) and improved the PFS (HR = 0.78, 95% CI = 0.71-0.85, p<0.00001;I2 = 0%) when compared with the placebo, and similar results was detected when compared with the single EGFR inhibition in terms of ORR (OR = 1.39, 95% CI = 1.12-1.74, p = 0.003; I2 = 30%) and PFS (HR = 0.73, 95% CI = 0.67-0.81, p<0.0001; I2 = 50%). No obvious difference was found between the combined inhibition therapy and single VEGF inhibition in term of ORR, however, combined inhibition therapy significantly decreased the PFS when compared to the single VEGF inhibition therapy (HR = 1.70, 95% CI = 1.34-2.17, p<0.0001; I2 = 50%). Besides, no significant difference was observed between the combined inhibition therapy and control therapy in term of OS (including placebo, single EGFR inhibition and single VEGF inhibition) (HR = 0.98, 95% CI = 0.92-1.04, p = 0.41; I2 = 0%).

**Conclusions:**

Combined inhibition therapy was superior to placebo and single EGFR inhibition in terms of ORR, PFS for advanced NSCLC, however, no statistical difference were found in term of OS. Besides, combined inhibition therapy was not superior to single VEGF inhibition in terms of ORR, PFS and OS. Therefore, combined inhibition therapy is recommended to treat advanced NSCLC patients.

## INTRODUCTION

Lung cancer is the leading cause of cancer-related death worldwide both in men and women, with 1.6 million new cases and 1.38 million deaths annually [1]. According to National Cancer Institute Surveillance, Epidemiology, and End Results (SEER) Program, non-small-cell lung cancer (NSCLC) accounts for about 85% of all invasive lung cancer among all cancer cases and the overall 5-year survival of patients with advanced NSCLC still remains approximately 17.4 % [2].

Unfortunately, around 57% patients with NSCLC have distant spread at the time of diagnosis, and a majority of them miss the chance to be offered surgery with curative intention [2]. The platinum-based therapy with or without targeted drugs becomes the main stream for the patients staged higher than IIIB, however, with high incidence of adverse effects [3]. Based on the treatment, although around 50-80% patients have rapid overall response rate (ORR), the rate of best response is low. Meanwhile, due to the disappointing progression free survival (PFS) and overall survival (OS), lots of patients have to receive the second-line treatment [4]. Therefore, more attention was paid to the targeted therapy.

The vascular endothelial growth factor (VEGF) is an important cancer marker and plays an crucial role in the tumor growth, invasion and metastasis [5], and gradually becomes a promising molecular target for the therapy of advanced NSCLC. Bevacizumab combined with carboplatin and paclitaxel chemotherapy has been approved for treat advanced NSCLC by Food and Drug Administration (FDA). Meanwhile, several trials have been conducted to explore the curative effect and toxicity of anti-VEGF drugs. The study conducted by Zhou et al presented that Bevacizumab significantly improved the PFS (median, 9.2 vs 6.5 months, respectively; hazard ratio (HR) = 0.40, 95% CI = 0.29-0.54, p < 0.001) and OS (median, 24.3 vs 17.7 months, respectively; HR = 0.68, 95% CI = 0.50-0.93, p = 0.0154) compared to the placebo in patients with advanced or recurrent NSCLC [6]. The epidermal growth factor receptor (EGFR) is a fatal cancer marker, and is involved with lots of intracellular pathways which promote cancer-cell proliferation, invasion, metastasis, and stimulate tumor-induced neovascularization [7–9]. The oral EGFR tyrosine kinase inhibitor (TKI), erlotinib, is approved by the FDA depending on extend overall survival (OS) in previously treated non-small-cell lung cancer [10]. The study conducted by Rosell et al reported that erlotinib significantly improved the PFS when compared to the chemotherapy (HR = 0.37, 95%CI = 0.25-0.54, p < 0.0001) [11].

However, drug resistance of targeted therapy is gradually increasing in clinical practice. Targeting multiple molecular pathways is a promising method to avoid the development of resistance and increase therapeutic effect. Hence, many clinical trials were carried out to explore the efficacy of combined VEGF and EGFR inhibition in advanced NSCLC. The study conducted by the Natale et al reported that the Vandetanib significantly improved the PFS compared to the Gefitinib, but no statistical difference was detected in term of OS [12]. Meanwhile, Boer et al covered that combined VEGF and EGFR inhibition could not obviously improved both PFS and OS [13]. Therefore, the comparative effectiveness of combined therapy inhibiting EGFR and VEGF pathways was controversial. A previous meta-analysis conducted by Ma et al only focused on the safety profile between the combined inhibition therapy and control therapy [14]. Hence, the aim of this study was to explore the comparative effectiveness between the combined therapy inhibiting EGFR and VEGF pathways and the control therapy (including placebo, single EGFR inhibition and single VEGF inhibition).

## MATERIALS AND METHODS

### Literature search strategy

A comprehensive search was conducted to identify the relevant studies in PubMed, EMBASE and the Cochrane library up to September 7, 2016. European Society of Medical Oncology (ESMO) and American Society of Clinical Oncology (ASCO) were also reviewed. The search strategy was (((“Bevacizumab” OR “Avastin” OR “Sunitinib” OR “Sutent” OR “Sorafenib” OR “Nexavar” OR “Pazopanib” OR “Votrient” OR “Cediranib” OR “Recentin” OR “Axitinib”) AND (“Erlotinib” OR “Gefitinib” OR “Cetuximab” OR “Panitumumab” OR “Lapatinib”)) OR (“Vandetanib” OR “Zactima”)) AND (“NSCLC” OR “non-small-cell lung cancer” OR “non-small-cell lung carcinoma”) AND (“RCT” OR “Randomized Controlled Trial”). All eligible studies were retrieved and inspected by reading the full text, and their reference lists were also checked to prevent missing studies.

### Inclusion criteria

Studies focusing on the comparisons of effectiveness between combined inhibition therapy and control therapy in treated patients with advanced NSCLC were eligible for inclusion. Included studies should meet all the following criteria: (i) published in English; (ii) reporting effectiveness of combined inhibition therapy and the control therapy (including placebo, single EGFR inhibition and single VEGF inhibition) in patients with advanced NSCLC; (iii) phase II/III randomized controlled trials (iv) enough data to calculate ORR, PFS or OS.

### Exclusion criteria

The exclusion criteria were as follows: (i) not phase II/III randomized controlled trials; (ii) ongoing studies; (iii) incomplete date; (iv) studies not within the field of interest of this study.

### Data extraction

As for each study, the following information was extracted: year of publication, trial phase, the first author's surname, the published journal, number of subjects, the percentage of male, median age, median PFS, median OS and treatment arm. Data extraction and information on study design, outcomes were performed by two independent reviewers (Wang H and Hui J) and disagreements were resolved by discussion and consensus with a third reviewer (Qi X).

### Statistical analysis

Pooled analyses were conducted by Review Manager 5.2. Dichotomous data were compared by OR. The survival data analysis was assessed by HR, which were directly obtained from the article or calculated by using previously published methods [15]. Forest plots were generated for graphical presentations, and heterogeneity among different studies was appraised by Q statistics and I2 estimates. Fixed-effects model was conducted to aggregate data if there were no statistical heterogeneity (I2 < 50%). However, when effects were heterogeneous (I2 > 50%), randomized effects model was carried out. Publication bias was examined with analyses described by Egger and Begg test with stata12.0. Influence analysis was employed to the study by stata12.0. The 95% CI for each result were computed.

## RESULTS

### Literature search

**Figure 1:**
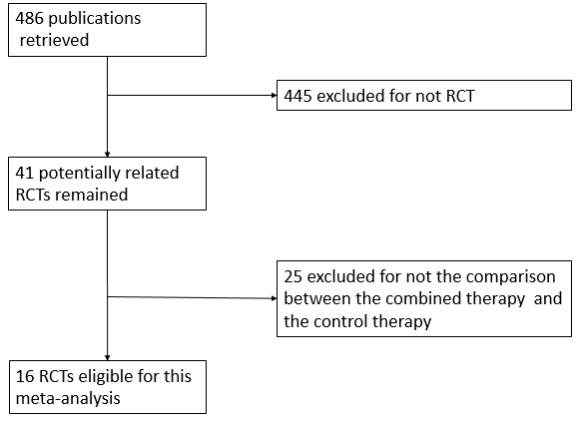
Flow diagram of study selection process

As shown in Figure 1, a total of 486 initial articles were retrieved. 445 articles were excluded for not RCTs. As for the 41 potentially related RCTs remained, 25 were excluded for not the comparison between the combined inhibition therapy and the control therapy. At last, 16 RCTs involved 7109 patients were eligible for this meta-analysis [12, 13, 16–29].

### Characteristics of included studies

The details characteristics of the included studies were listed in Table 1. The sixteen included studies were made up of six phase III RCTs [13, 19, 21, 22, 24, 25] and ten phase II RCTs [12, 16–18, 20, 23, 26–29]. Eleven studies [12, 13, 16, 17, 19, 21–25, 27] focused on the treatment of previously treated patients with advanced NSCLC, and five studies [18, 20, 26, 28, 29] focused on the first-line treatment. The median age of patients ranged from 58 to 68 years old. Besides, the median PFS varied from 7.2 weeks to 16.0 months, and the OS varied from 6.6 months to 16.4 months. ORR was reported in all eligible studies [12, 13, 16–29]. PFS was reported in fifteen studies [12, 13, 16–19, 21–29] and OS were reported in fourteen studies [13, 16–27, 29].

**Table 1:** Characteristics of the included studies

Study name	Published	Randomized	Patients	Published	Male (%)	Median age	Median	Median	Treatment
	year	clinical trial	(n)	journal	(Arm-1 vs Arm-2)	(years)	PFS	OS	
Herbst et al[16]	2007	Phase II	120	J Clin Oncol	43.6 vs 57.5	68 vs 63.5	4.4 vs 4.8m	13.7 vs 12.6m	Arm-1: Bevacizumab + Erlotinib.
									Arm-2: Bevacizumab +Chemotherapy
Heymach et al(1)	2007	Phase II	83	J Clin Oncol	50 vs 66	61 vs 58	18.7vs12.0w	13.1 vs 13.4m	Arm-1: Vandetanib (100 mg) +Docetaxel.
[17]									Arm-2: Placebo+Docetaxel
Heymach et al(2)	2007	Phase II	85	J Clin Oncol	57 vs 66	60 vs 58	17.0vs12.0w	7.9 vs 13.4m	Arm-1: Vandetanib (300 mg) +Docetaxel.
[17]									Arm-2: Placebo+Docetaxel
Natale et al[12]	2009	Phase II	168	J Clin Oncol	58 vs 61	63 vs 61	11.0 vs 8.1w	NA	Arm-1: Vandetanib Arm-2: Gefitinib
Herbst et al[19]	2010	Phase III	1391	Lancet Oncol	72 vs 68	59 vs 59	4.0 vs3.2m	10.6 vs 10.0m	Arm-1: Vandetanib+Docetaxel.
									Arm-2: Placebo+Docetaxel
Spigel et al[23]	2011	Phase II	168	J Clin Oncol	56 vs 47	65 vs 65	3.38vs1.94m	7.62 vs 7.23m	Arm-1: Sorafenib+Erlotinib.
									Arm-2: Placebo+Erlotinib
Natale et al[22]	2011	Phase III	1240	J Clin Oncol	61 vs 64	61 vs 61	2.6 vs 2.0m	6.9 vs 7.8m	Arm-1: Vandetanib
									Arm-2: Erlotinib
Herbst et al[21]	2011	Phase III	636	Lancet	54 vs 54	64.8 vs 65.0	3.4 vs 1.7m	9.3 vs 9.2m	Arm-1: Bevacizumab+Erlotinib.
									Arm-2: Placebo+Erlotinib
Boer et al[13]	2011	Phase III	534	J Clin Oncol	62 vs 62	60 vs 60	17.6vs11.9w	10.5 vs 9.6m	Arm-1: Vandetanib+Pemetrexed.
									Arm-2: Placebo+Pemetrexed
Lee et al[24]	2012	Phase III	924	J Clin Oncol	47 vs 48	60 vs 60	1.9 vs 1.8m	8.5 vs 7.8m	Arm-1: Vandetanib
									Arm-2: Placebo
Scagliotti et al[25]	2012	Phase III	960	J Clin Oncol	61.9 vs 59.2	61 vs 61	3.6 vs 2.0m	9.0 vs 8.5m	Arm-1: Sunitinib +Erlotinib.
									Arm-2: Placebo+Erlotinib
Groen et al[27]	2013	Phase II	132	ANN ONCOL	39 vs 45	59 vs 61	2.8 vs 2.0m	8.2 vs 7.6m	Arm-1: Sunitinib +Erlotinib.
									Arm-2: Placebo+Erlotinib
Seto et al[28]	2014	Phase II	152	Lancet Oncol	40 vs 34	67 vs 67	16 vs 9.7m	NA	Arm-1: Bevacizumab+Erlotinib.
									Arm-2: Placebo+Erlotinib
Ciuleanua et al[26]	2013	Phase II	124	Lung Cancer	59 vs 59	61 vs 58	18.4vs25.0w	16.4 vs NAm	Arm-1: Bevacizumab+Erlotinib.
									Arm-2: Bevacizumab+ Chemotherapy
Gridelli et al [20]	2011	Phase II	60	Ann Oncol	59 vs 65	76 vs 74	NA	12.6 vs 6.55m	Arm-1: Erlotinib + Sorafenib
									Arm-2: Gemcitabine +Sorafenib
Heymach et al[18]	2008	Phase II	108	J Clin Oncol	70 vs 71	60 vs 59	24.0vs23.0w	10.2 vs 12.6m	Arm-1:Vandetanib+Paclitaxel+ Carboplatin
									Arm-2:Placebo+ Paclitaxel+ Carboplatin
Thomas et al[29]	2015	Phase II	224	Eur Respir J	56.8 vs 55.8	62 vs 60	3.5vs 6.9m	12.6 vs 17.7m	Arm-1: Erlotinib+ Bevacizumab
									Arm-2: Chemotherapy +Bevacizumab

m:month; NA: not available; ANN ONCOL: Annals of Oncology; Eur Respir J:european respiratory journal; J Clin Oncol: journal of clinical oncology; Lancet Oncol: Lancet Oncology.

Five studies compared vandetanib with placebo [13, 17–19, 24], and seven studies [12, 21–23, 25, 27, 28]made the comparison between the combined inhibition therapy and single EGFR inhibition therapy. Four studies [16, 20, 26, 29] focused on the efficacy comparison between the combined inhibition therapy and single VEGF inhibition. In addition, the study conducted by Heymach et al was divided into two sections according to the different dose of vandetanib [16].

### Meta-analyses of ORR

**Figure 2:**
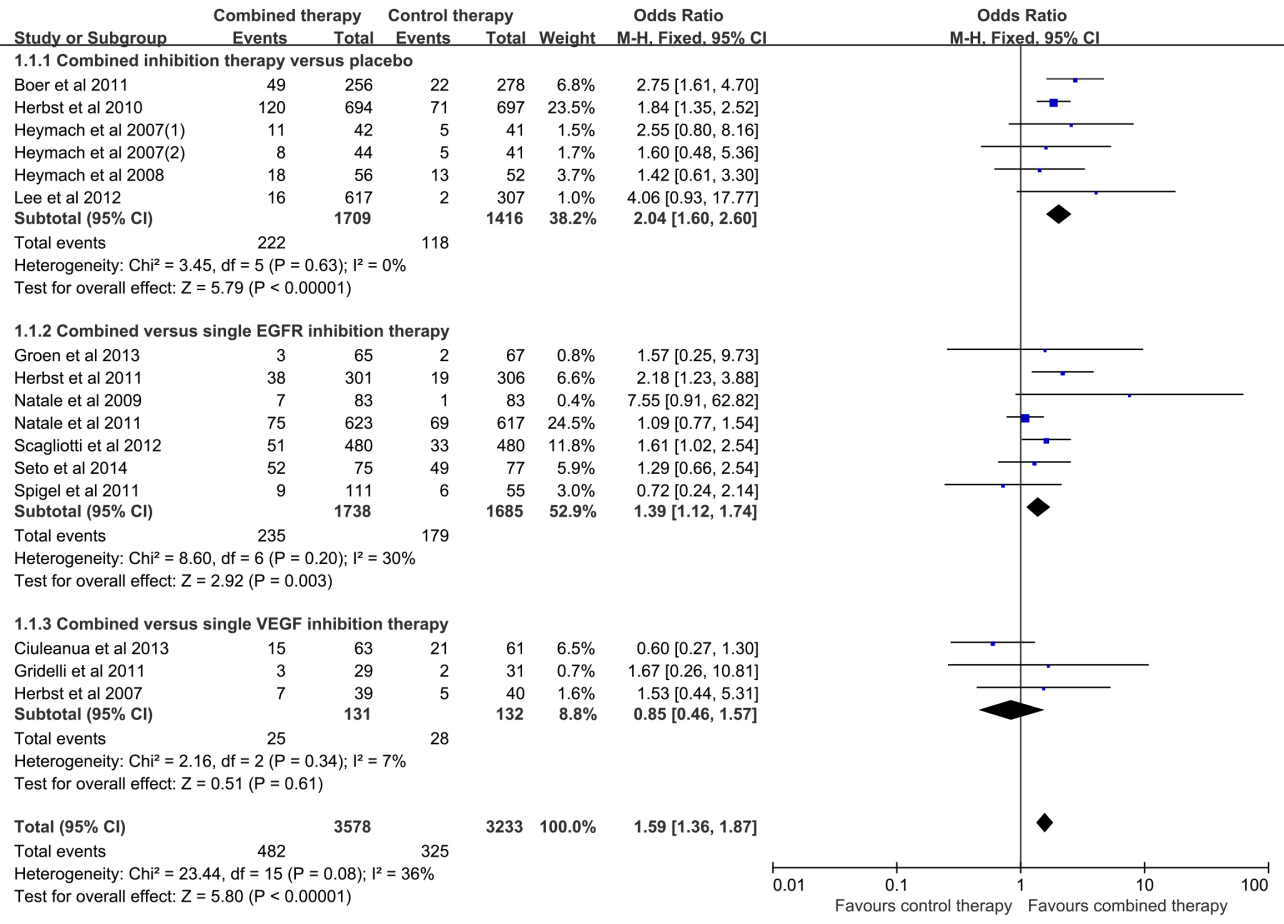
Meta-analysis of overall response rate

All the included studies reported the ORR, however, the study conducted by the Thomas et al was excluded for significantly increased heterogeneity. As listed in Figure 2, fixed effect model was used for no heterogeneity existence (I2 = 36%, p = 0.08), and the results of the meta-analysis revealed that the combined inhibition therapy significantly increased the overall response rate when compared to the control therapy (OR = 1.59, 95% CI = 1.36-1.87, p < 0.00001).Subgroup analysis was performed based on the control therapy. The results presented that higher ORR was detected in the combined inhibition therapy group when compared to the placebo (OR = 2.04, 95% CI = 1.60-2.60, p < 0.00001; I2 = 0%) and the single EGFR inhibition therapy (OR = 1.39, 95% CI = 1.12-1.74, p = 0.003; I2 = 30%) respectively. However, no significant difference was found between the combined inhibition therapy and the single VEGF inhibition therapy (OR = 0.85, 95% CI = 0.46-1.57, p = 0.61; I2 = 7%). As listed in Table 2, in the previously treated patients, the ORR was significantly increased in the combined inhibition therapy group when compared with the control group (OR = 1.70, 95%CI = 1.44-2.02, p < 0.00001; I2 = 34%). However, as first-line treatment, no significant difference was detected between the combined inhibition therapy and control therapy (OR = 1.07, 95% CI = 0.70-1.62, p = 0.77; I2 = 3%). Besides, there was no bias among all included studies (Begg test, p = 0.499; Egger test, p = 0.665), and no decisive effect according to the influence analysis conducted by Stata12.0 (Supplementary Figure 1).

**Table 2:** Main other results of the study

**ORR**					
**First line**		**Included studies**	**OR 95% CI**	**p value**	**I^2^**
	Combined inhibition therapy versus placebo	1	1.42 [0.61, 3.30]	0.41	NA
	Combined versus single EGFR inhibition therapy	1	1.29 [0.66, 2.54]	0.46	NA
	Combined versus single VEGF inhibition therapy	2	0.70 [0.34, 1.43]	0.33	0%
	Total	4	1.07 [0.70, 1.62]	0.77	3%
**Second or more line**					
	Combined inhibition therapy versus placebo	4	2.11 [1.64, 2.72]	<0.00001‡	0%
	Combined versus single EGFR inhibition therapy	6	1.41 [1.11, 1.78]	0.005‡	42%
	Combined versus single VEGF inhibition therapy	1	1.53 [0.44, 5.31]	0.5	NA
	Total	11	1.70 [1.44, 2.02]	<0.00001‡	34%
**Total**		15	1.59 [1.36, 1.87]	<0.00001‡	36%
**PFS**					
**First line**		**Included studies**	**HR 95% CI**	**p value**	**I^2^**
	Combined inhibition therapy versus placebo	1	0.76 [0.51, 1.13]	0.18	NA
	Combined versus single EGFR inhibition therapy	1	0.54 [0.36, 0.81]	0.003‡	NA
	Combined versus single VEGF inhibition therapy	2	1.88 [1.45, 2.44]	<0.0001‡	0%
	Total	4	1.10 [0.57, 2.13]	0.77	90%
**Second or more line**					
	Combined inhibition therapy versus placebo	4	0.78 [0.71, 0.85]	<0.00001‡	0%
	Combined versus single EGFR inhibition therapy	5	0.75 [0.68, 0.82]	<0.0001‡	48%
	Combined versus single VEGF inhibition therapy	1	0.95 [0.51, 1.78]	0.88	NA
	Total	10	0.76 [0.71, 0.82]	<0.00001‡	19%
**Total**		14	0.83 [0.72, 0.96]	=0.01‡	77%
**OS**					
**First line**		**Included studies**	**HR 95% CI**	**p value**	**I^2^**
	Combined inhibition therapy versus placebo	1	1.15 [0.75, 1.76]	0.52	NA
	Combined versus single VEGF inhibition therapy	3	1.28 [0.99, 1.66]	0.06	0%
	Total	4	1.24 [1.00, 1.55]	0.05	0%
**Second or more line**					
	Combined inhibition therapy versus placebo	4	0.93 [0.84, 1.03]	0.16	0%
	Combined versus single EGFR inhibition therapy	5	0.97 [0.89, 1.05]	0.48	0%
	Combined versus single VEGF inhibition therapy	1	1.12 [0.60, 2.09]	0.72	NA
	Total	10	0.96 [0.90, 1.02]	0.16	0%
**Total**		14	0.98 [0.92, 1.04]	0.41	0%

ORR, overall response rate; PFS, progression-free survival; OS overall survival; NA, not applicable; ‡ p < 0.05, the difference is significant.

### Meta-analyses of PFS

**Figure 3:**
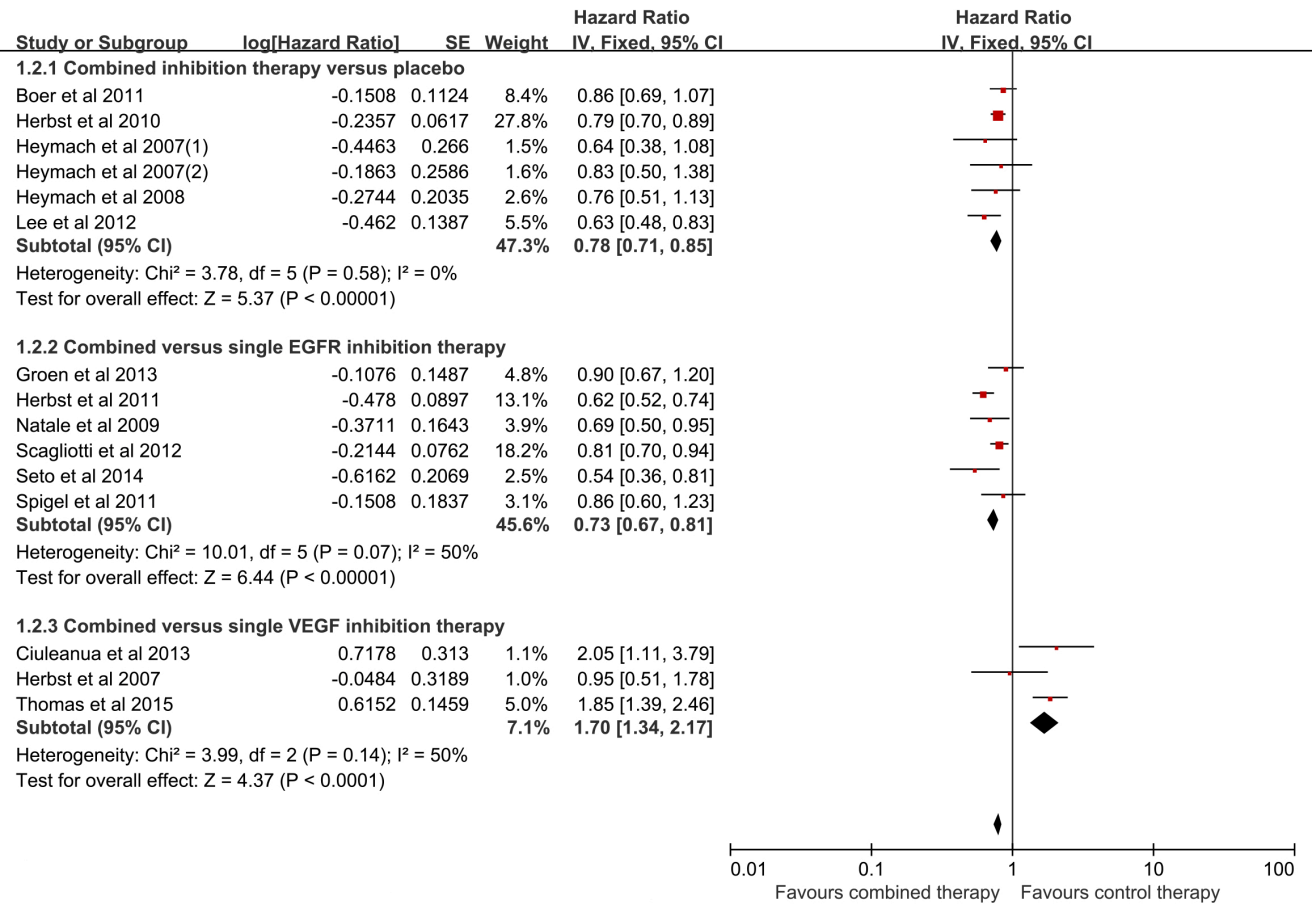
Meta-analysis of progression free survival

Fifteen of the eligible studies covered the PFS, but the study conducted by the Natale et al published in 2011 was excluded for the significant increase of heterogeneity. Therefore, fourteen studies were engaged into the meta-analysis of PFS. As shown in Figure 3, on account of no heterogeneity, fixed effect model was employed (I2 = 0%, p = 0.58). The results indicated that the combined inhibition therapy significantly improved PFS compared with the placebo (HR = 0.78, 95% CI = 0.71-0.85, p < 0.00001). Besides, the PFS was remarkably improved in combined inhibition therapy group compared with single EGFR inhibition therapy group (HR = 0.73, 95%CI = 0.67-0.81, p < 0.0001, I2 = 50%), while was decreased compared with single VEGF inhibition therapy group (HR = 1.70, 95%CI = 1.34-2.17, p < 0.0001, I2 = 50%). As for the previously treated patients, the combined inhibition therapy distinctly improved the PFS when compared to the control therapy, with a random model (HR = 0.76, 95%CI = 0.71-0.82, p < 0.00001; I2 = 19%) (Table 2). In the first-line treatment, no statistical differences was observed between the combined inhibition therapy and the control therapy, using a random model (HR = 1.10, 95%CI = 0.57-2.13, p = 0.77; I2 = 90%) (Table 2). No bias among all included studies was detected (Begg test, p = 0.428; Egger test, p = 0.578). There was no decisive effect according to the influence analysis conducted by the Stata 12.0 (Supplementary Figure 2).

### Meta-analyses of OS

**Figure 4:**
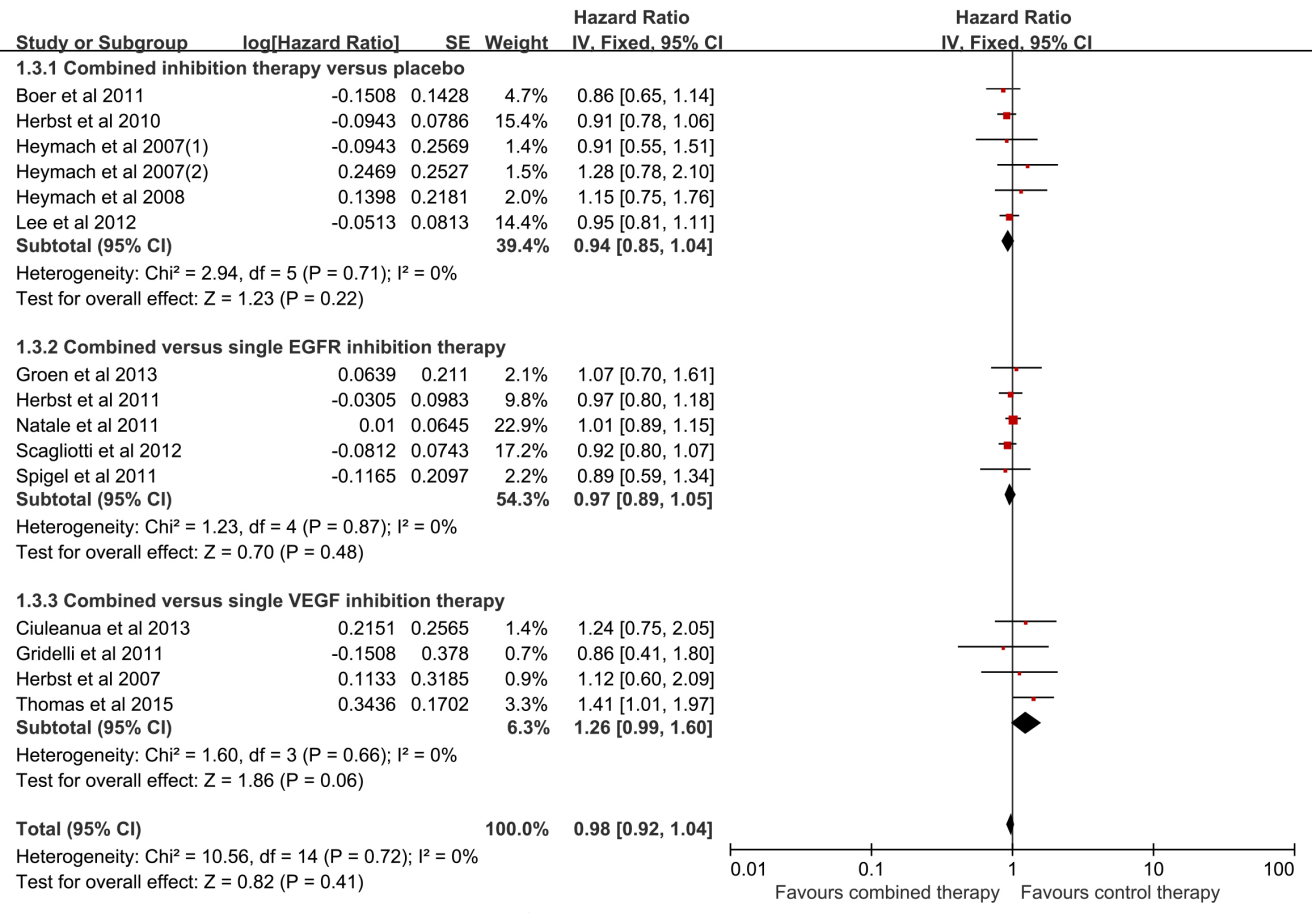
Meta-analysis of overall survival

Fourteen studies reported the OS. As listed in Figure 4, there was no heterogeneity among the included studies (I2 = 0%, p = 0.72), and no significant difference was observed between the combined inhibition therapy and the control therapy (HR = 0.98, 95% CI = 0.92-1.04, p = 0.41). With regard to subgroup analysis, no significant difference was detected between the combined inhibition therapy and placebo (HR = 0.94, 95% CI = 0.85-1.04, p = 0.22; I2 = 0%), similar results were yielded when compared with the single EGFR inhibition therapy (HR = 0.97, 95%CI = 0.89-1.05, p = 0.48; I2 = 0%) and single VEGF inhibition therapy (HR = 1.26, 95%CI = 0.99-1.60, p = 0.06; I2 = 0%). As shown in Table 2, no statistical significant was observed between the combined inhibition therapy and control therapy in the previously treated patients (HR = 0.96, 95%CI = 0.90-1.02, p = 0.16; I2 = 0%), and similar result was detected for the previously untreated patients (HR = 1.24, 95%CI = 1.00-1.55, p = 0.05; I2 = 0%). There was no bias among the included studies (Begg test, p = 0.276; Egger test, p = 0.146). No decisive effect was observed according to the influence analysis (Supplementary Figure 3).

## DISSCUSSION

Combined therapy inhibiting EGFR and VEGF pathways is becoming a promising method to improve the monotherapy resistance in clinical practice. Hence, many phase II and phase III were carried out to explore the curative effect between the combined inhibition therapy and control therapy (including placebo, single EGFR inhibition and single VEGF inhibition) [12, 13, 16–28]. The study conducted by Lee et al covered that vandetanib significantly increased PFS compared to the placebo, however, no statistical difference was observed in terms of OS [24], and Herbst et al reported similar result [19]. Nevertheless, Boer et al reported opposite outcomes that no obvious difference was detected in term of PFS and OS [13]. As for the comparison between the combined and single EGFR inhibition therapy in previously treated patients, Groen et al declined that no significant difference were found in term of PFS [27], however, Herbst et al indicated improved PFS in combined inhibition therapy group. [21]. Therefore, it was suggested that dispute really existed in this filed.

In our study, the results revealed that combined inhibition therapy obviously increased the ORR when compared to the control therapy, similar results were detected when compared to the placebo and single EGFR inhibition in the subgroup analysis. No statistical difference was observed when compared to single VEGF inhibition in the subgroup analysis. And the result indicated that the previously treated patients had a better ORR in combined inhibition therapy group than control therapy group (including placebo, single EGFR inhibition and single VEGF inhibition), and similar results were detected in the subgroup analysis. However, no significant difference was observed in the previously untreated patients. As for the PFS, our study revealed that combined inhibition therapy prolonged the PFS compared with the control group (including placebo, single EGFR inhibition and single VEGF inhibition). And similar results were detected between combined therapy and placebo or single EGFR inhibition in subgroup analysis. However, no statistical significant was observed between the combined inhibition therapy and the single VEGF inhibition. Besides, the combined inhibition therapy clearly prolonged the PFS when compared to both the single EGFR and VEGF inhibition in the first-line treatment. Compared with the placebo and single EGFR inhibition, the combined inhibition therapy significantly improved the PFS in the treatment of previously treated patients with advanced NSCLC. In term of OS, no matter in first-line treatment or second-line treatment, no significant difference were found between the combined inhibition therapy and control therapy (including placebo, single EGFR inhibition and single VEGF inhibition), which was different from the previous meta-analysis [30]. And similar results were detected in the subgroup analysis.

In our study, the combined inhibition therapy had better ORR and longer PFS when compared to the placebo and EGFR inhibition therapy. A meta-analysis conducted by Ma et al yielded that no statistical difference were found between the combined inhibition therapy and placebo in term of all grades adverse effects [14]. Therefore, it is indicated that the combined inhibition therapy may be a better option for the patients with advanced NSCLC, especially for the previously EGFR inhibition treated patients. In addition, efficacy in term of ORR between the combined inhibition therapy and single VEGF inhibition was equivalent. Moreover, the combined inhibition therapy might decrease the PFS when compared to the single VEGF inhibition, especially in first-line treatment, which might be explained that some included studies of the control therapy was combined chemotherapy not the targeted therapy [20, 26, 29]. Because of the limited included studies, subgroup analysis was not concluded. Therefore, more clinical trials should be carried out to explore the comparative efficacy between them. It must now be said that combined inhibition therapy had no obvious effect on the OS when compared to the control therapy (including placebo, single EGFR inhibition and single VEGF inhibition).

A previous meta-analysis conducted by Rai et al covered that combined inhibition therapy significantly improved the ORR, PFS and OS [30] but it only consisted of seven studies and only focused on the previously treated patients with advanced NSCLC. Ma et al covered the comparison between the combined inhibition therapy and control therapy (including placebo, single EGFR inhibition and single VEGF inhibition), nevertheless, their meta-analysis only focused on the safety profile, not the ORR, PFS or OS. Besides, our study first reported the comparison between the combined inhibition therapy and placebo.

The highlighted strength of our meta-analysis as follows: Firstly, it focused on the comparative efficacy between the combined inhibition therapy and control therapy (including placebo, single EGFR inhibition and single VEGF inhibition). Secondly, all included studies were phase II or phase III RCTs, and most of them were multicenter trials with relatively large population. Thirdly, the comparison was divided into multiple subgroup analysis and the analysis was comprehensive.

Some limitations of our study should be considered. Firstly, some included studies were not adequate and well-controlled studies, which might influence the results [20, 26, 29]. Secondly, with significant heterogeneity in some analyses, the random model was used and might affect the accuracy of the study. Thirdly, because of all the datum was extracted from the published papers the individual data, such as drug dose and the prior therapy, was unavailable.

In conclusion, combined inhibition therapy was superior to placebo and single EGFR inhibition in terms of ORR, PFS for advanced NSCLC, however, no statistical difference were found in term of OS. Besides, combined inhibition therapy was not superior to single VEGF inhibition in terms of ORR, PFS and OS. Therefore, combined inhibition therapy is recommended to treat advanced NSCLC patients.

## SUPPLEMENTARY MATERIALS FIGURES





## Abbreviation

NSCLC: non-small-cell lung cancer
CENTRAL: Cochrane Central Register of Controlled Trials
ASCO: American Society of Clinical Oncology
ESMO: European Society of Medical Oncology
RCT: randomized controlled trial
ORR: overall response rate
PFS: progression free survival
OS: overall survival
VEGF: vascular endothelial growth factor
FDA: Food and Drug Administration
EGFR: epidermal growth factor receptor
TKI: tyrosine kinase inhibitor.
