# Supplementary material for: Comparative effectiveness of combined therapy inhibiting EGFR and VEGF pathways in patients with advanced non-small-cell lung cancer: a meta-analysis of 16 phase II/III randomized trials
Source: Oncotarget. 2016 Sep 27;8(4):7014–24. doi: 10.18632/oncotarget.12294 (PMC5351687; doi:10.18632/oncotarget.12294)
Supplement: Supplementary file 1 [file oncotarget-08-7014-s001.pdf]

Comparative effectiveness of combined therapy inhibiting EGFR and VEGF pathways in patients with advanced non-small-cell lung cancer: a meta-analysis of 16 phase II/III randomized trials

Supplementary Material

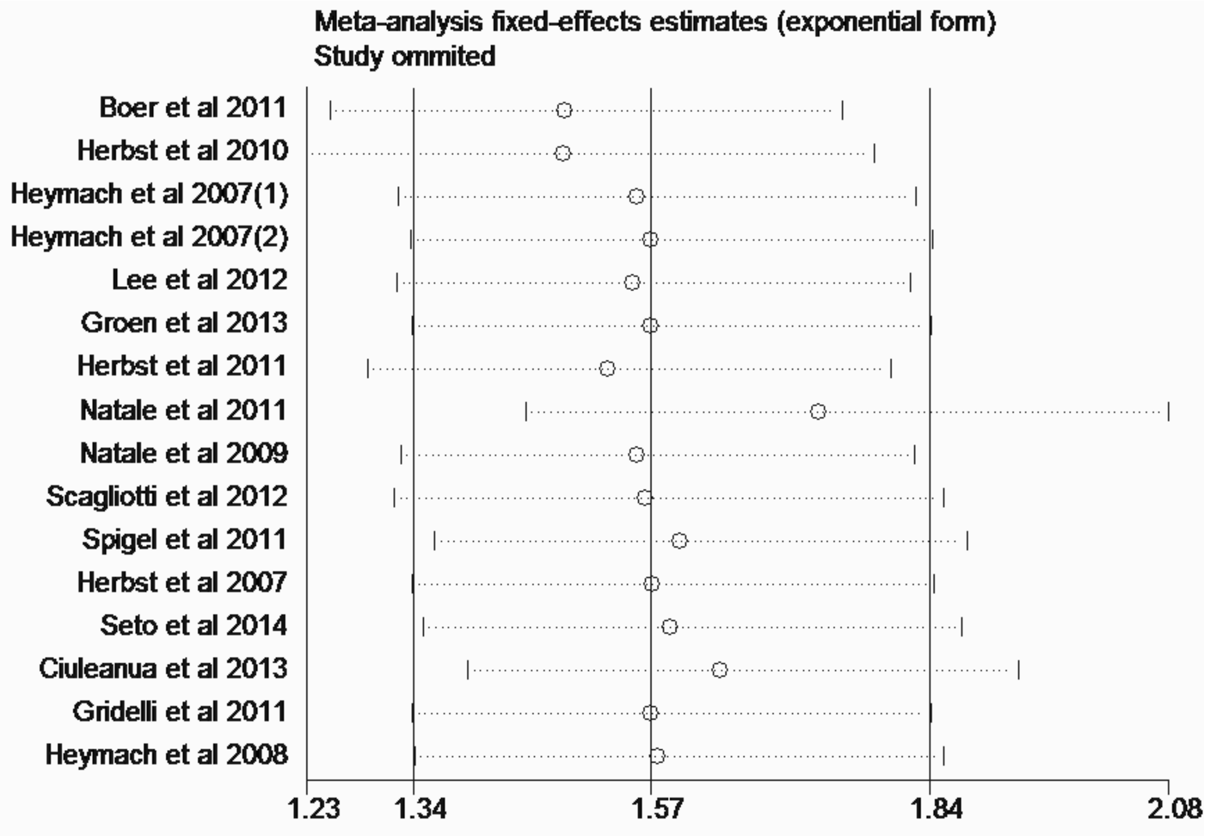

Supplementary Figure 1 Influence analysis of overall response rate.

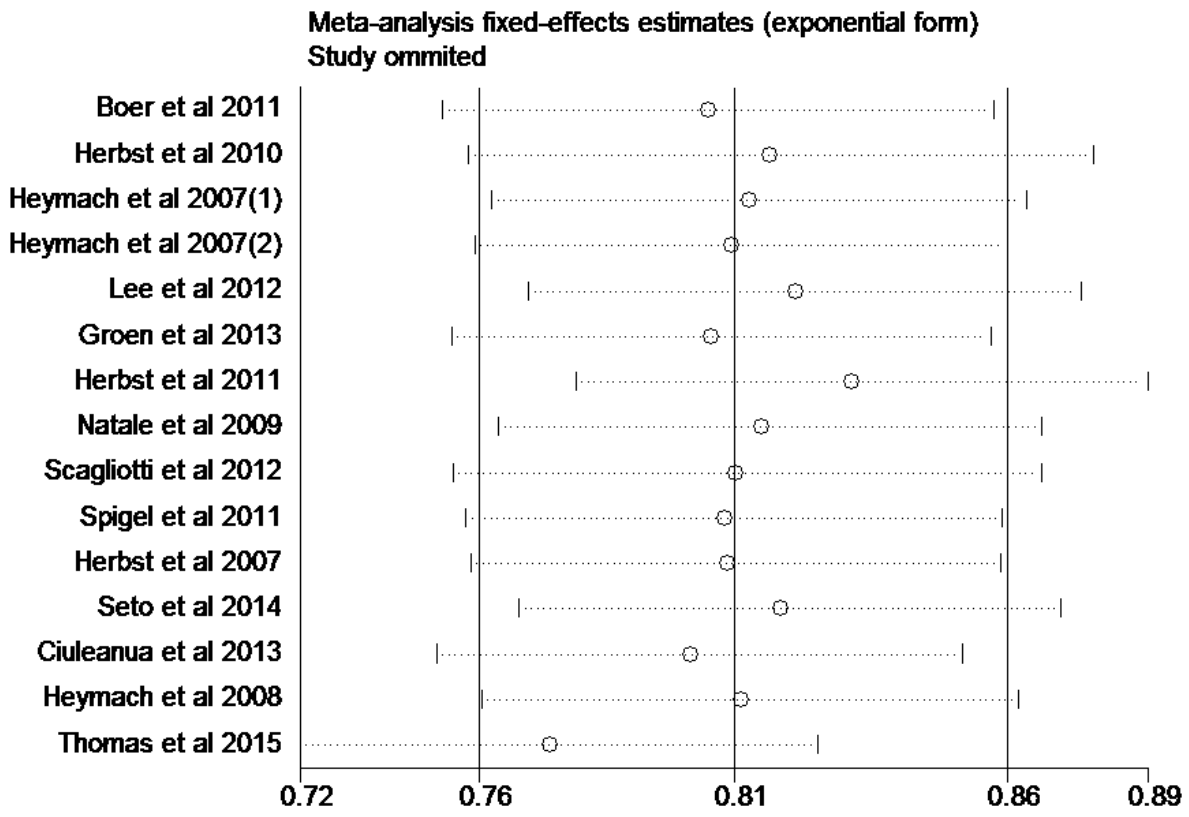

Supplementary Figure 2 Influence analysis of progression free survival.

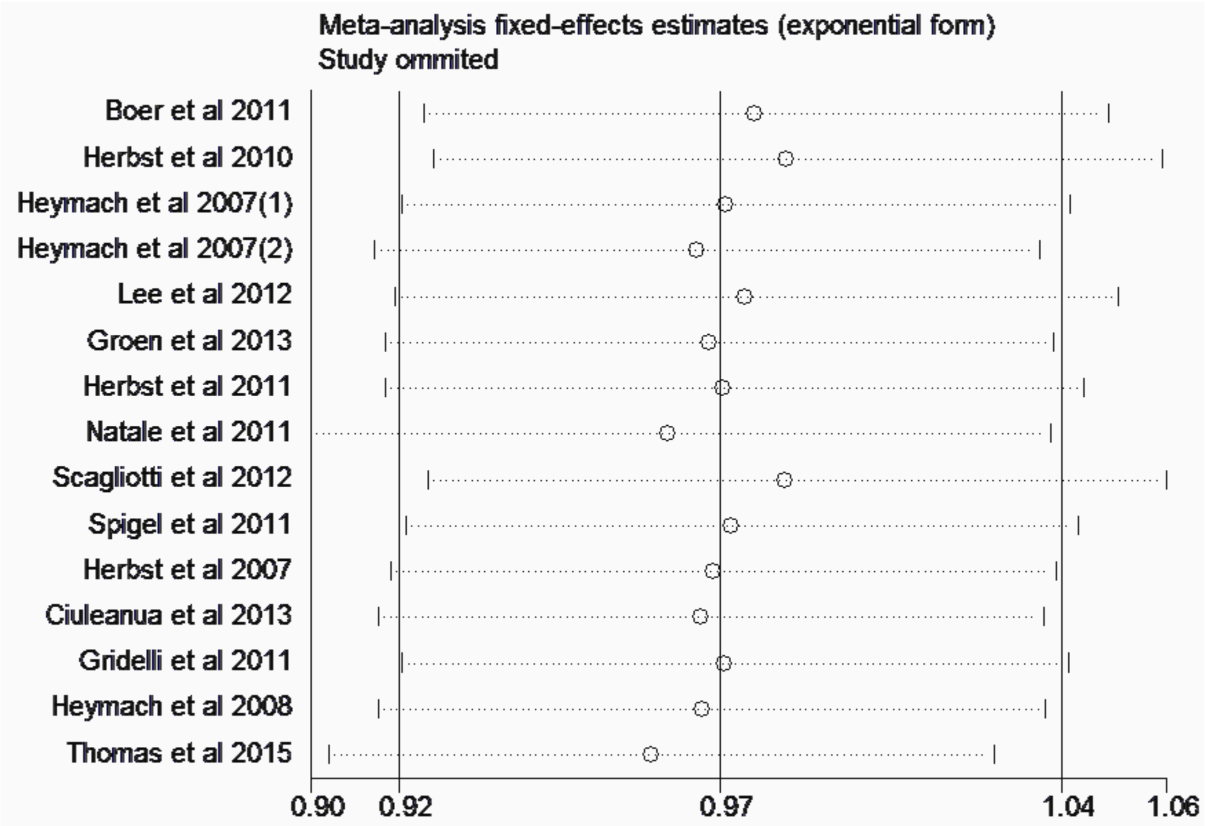

Supplementary Figure 3 Influence analysis of overall survival.
